# A Systematic Study of the Impact of Estrogens and Selective Estrogen Receptor Modulators on Prostate Cancer Cell Proliferation

**DOI:** 10.1038/s41598-020-60844-3

**Published:** 2020-03-04

**Authors:** Camille Lafront, Lucas Germain, Cindy Weidmann, Étienne Audet-Walsh

**Affiliations:** 1 Department of molecular medicine, Faculty of Medicine, Université Laval, Québec City, G1V 0A6 Canada; 2 Endocrinology - Nephrology Research Axis, Centre de recherche du CHU de Québec - Université Laval, Québec City, Canada; 3 Centre de recherche sur le cancer (CRC) of Université Laval, Québec City, Canada; 4 Department of biochemistry, microbiology and bioinformatics, Faculty of Sciences and Engineering, Université Laval, Québec City, G1V 0A6 Canada

**Keywords:** Breast cancer, Prostate cancer, Cancer, Endocrinology, Molecular medicine, Oncology, Urology

## Abstract

The estrogen signaling pathway has been reported to modulate prostate cancer (PCa) progression through the activity of estrogen receptors α and β (ERα and ERβ). Given that selective estrogen receptor modulators (SERMs) are used to treat breast cancer, ERs have been proposed as attractive therapeutic targets in PCa. However, many inconsistencies regarding the expression of ERs and the efficacy of SERMs for PCa treatment exist, notably due to the use of ERβ antibodies lacking specificity and treatments with high SERM concentrations leading to off-target effects. To end this confusion, our objective was to study the impact of estrogenic and anti-estrogenic ligands in well-studied *in vitro* PCa models with appropriate controls, dosages, and ER subtype-specific antibodies. When using physiologically relevant concentrations of nine estrogenic/anti-estrogenic compounds, including five SERMs, we observed no significant modulation of PCa cell proliferation. Using RNA-seq and validated antibodies, we demonstrate that these PCa models do not express ERs. In contrast, RNA-seq from PCa samples from patients have detectable expression of ERα. Overall, our study reveals that commonly used PCa models are inappropriate to study ERs and indicate that usage of alternative models is essential to properly assess the roles of the estrogen signaling pathway in PCa.

## Introduction

In the context of prostate cancer (PCa), the androgen receptor (AR) has many oncogenic functions such as increasing the proliferation and survival of cancer cells^1^. Furthermore, it is also now known that AR is an important regulator of metabolic pathways that sustain aberrant proliferation of PCa cells^2–6^. Accordingly, current hormonal treatments target this receptor with the objective of inhibiting its functions, with anti-androgens or androgen deprivation therapies^1,7^. However, despite a positive response to these treatments initially, progression of the disease to castration-resistant PCa (CRPC) is mostly inevitable^1,7^. This highlights the need to find novel approaches for the treatment of PCa.

Estrogens and the most active form estradiol (E_2_) are naturally produced from androgens through steroidogenesis and have been linked to PCa evolution, as reviewed recently by Dobbs *et al*.^8^. Indeed, it has been demonstrated in murine models and in human patients that increased levels of estrogens were positively correlated with the aggressiveness of PCa^9–15^, and that estrogen synthesis increases in cancer cells during disease progression^16–20^. This modulation of cancer evolution is thought to be caused by the activity of the estrogen receptors ERα and ERβ, two transcription factors that are both essential for the normal development of the prostate^21–23^. The actual model supports that ERα has oncogenic functions, as seen in murine models where its activation leads to an increased proliferation of cancer cells^24–26^; on the other hand, ERβ is thought to act as a tumor suppressor since its loss promotes prostate hyperplasia and the development of the disease^21,27–30^. Further supporting this model, ERα expression is reported to be increased and ERβ to be decreased during PCa progression^31–37^. These findings suggest that targeting the estrogen signaling pathway could be a viable therapeutic avenue for the management of PCa and CRPC.

Several anti-estrogen therapies are currently in use for the treatment of ERα-positive breast cancer tumors^38^. These drugs include tamoxifen, raloxifene, and toremifene, and are now known to be selective estrogen receptor modulators (SERMs). As their name implies, they have the interesting capacity to be antagonist or agonist of the ERs in a tissue-specific manner; for example, tamoxifen is an antagonist of ERα in the mammary gland and breast cancer cells, but is an agonist in other peripheric tissues, such as the bone, thus limiting adverse side-effects of estrogen blockade throughout the whole body^39^. Importantly in the breast cancer field, assessing ERα expression levels in patient tumors is always performed prior to the selection of anti-estrogen therapies, as the presence of the receptor is tightly linked to the patient’s response. In the context of PCa, a few clinical studies using these compounds were conducted with limited sample sizes, and both null and positive responses were observed^7,40–45^. However, no initial molecular characterization have been performed in these studies, such as ERα and ERβ expression or activity in prostate tumors, explaining—at least in part—the discrepancies and the heterogeneity in patient responses.

Moreover, not all *in vitro* PCa models are appropriate to study ER functions, but they have still been inconsistently used in this context. For example, it has been known for decades that LNCaP cells—the most widely used human PCa model—have a mutated AR that can be activated by E_2_ in addition to androgens^46,47^ and have low, if any, expression of both ERs^48,49^. Nevertheless, several groups used this model to study E_2_ impact on PCa cell proliferation and survival^50–52^. In addition, the lack of specific ERβ antibody, as clearly described recently^48,53,54^, has also lead to controvorsies in the literature regarding which PCa cell line models express or not ERβ. Finally, specific ligands for both ERs exist, such as PPT for ERα and DPN for ERβ. Yet, precise dosages have to be used to keep this specificity, as higher concentrations will lead to dual activation of ERs or modulation of other pathways. For example, the EC_50_ of DPN is of 66 nM and 0.85 nM for ERα and ERβ (Table 1), respectively, and has been used at 100 nM and 1000 nM in previous studies as an “ERβ-specific ligand”^55–58^. The same issue has occurred for the ERα agonist PPT, where its EC_50_ is of 0.2 nM and 82 nM for ERα and ERβ (Table 1), respectively, but has been used at a concentration of 100 nM^57–59^. Likewise, high concentrations used for SERMs treatment can have numerous impacts on other receptors than ERs. For example, 4-hydroxytamoxifen, an active metabolite of tamoxifen, has an IC_50_ of approximately 3.3 nM for ERα and ERβ (Table 1), but if used at concentrations higher than 90 nM, it also inhibits the estrogen-related receptor ERRγ, another transcription factor member of the nuclear receptor family^60^. It is thus essential to use appropriate drug dosages in order to solely modulate ERs’ activity.

**Table 1:** Compound description with all the EC/IC50.

Compounds	Action in PCa cells	EC_50_/IC_50_^a^	Treatment concentration
PPT	Agonist of ERα	ERα: EC_50_ = 0.2 nM ERβ: EC_50_ = 82 nM	1 nM
DPN	Agonist of ERβ	ERα: EC_50_ = 66 nM ERβ: EC_50_ = 0.85 nM	4.25 nM
Fulvestrant	Antagonist of both ERs	ERα: IC_50_ = 0.47 nM ERβ: IC_50_ = 3.8 nM	19 nM
4-Hydroxytamoxifen (SERM)	Unsure^b^	ERs: IC_50_ = 3.3 nM	10 nM
Raloxifene (SERM)	Unsure^b^	ERs: IC_50_ = 2.9–5.7 nM	28.5 nM
Toremifene (SERM)	Unsure^b^	ERs: IC_50_ = 1,000 nM	1,000 nM
Bazedeoxifene (SERM)	Unsure^b^	ERα: IC_50_ = 26 nM ERβ: IC_50_ = 99 nM	495 nM
Lasofoxifene (SERM)	Unsure^b^	ERα: IC_50_ = 1.08 nM ERβ: IC_50_ = 4.41 nM	22.05 nM

^a^IC_50_ and EC_50_ were retrieved from supplier’s websites: Santa Cruz Biotechnology, Tocris, ApexBio, and MedChemExpress.

^b^SERMs are mostly believed to be antagonists in PCa cells, but this is mostly based on experiments performed in the same *in vitro* models as used in the current study.

Overall, it is still not clear which PCa models represent a good model to study ERs functions, what is the impact of activating ERα and/or ERβ on PCa cell proliferation, and if SERMs and the pure antiestrogen fulvestrant can be used to block PCa cell proliferation. The aim of our study was to perform a systematic investigation of the impact of treatments with natural estrogen, specific ERα and ERβ ligands, and SERMs, at specific concentrations, on PCa cell proliferation.

## Results

### ERs mRNA and protein expression levels in breast cancer and PCa models

**Figure 1:**
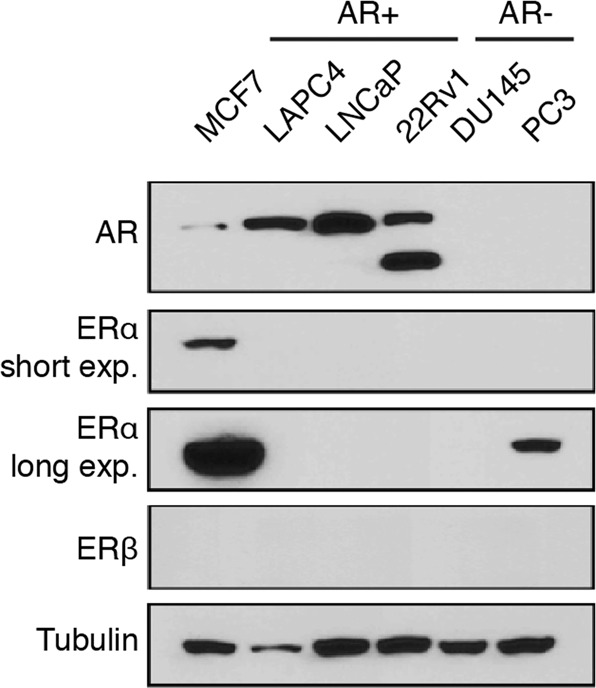
Weak estrogen receptors protein expression in PCa cell lines. Protein expression of AR, ERα, and ERβ in MCF7, LNCaP, LAPC4, 22Rv1, DU145 and PC3. α-tubulin was used as a loading control. No bands were detectable for ERβ at any exposure.

First, we assessed the protein expression levels of ERα and ERβ in our PCa models using recently validated antibodies^48,61,62^. We used as control the human breast cancer cell line MCF7, which showed high expression levels of ERα (as expected), no expression of ERβ and weak but detectable expression of AR (Fig. 1). All human AR-positive PCa cell lines (LNCaP, LAPC4 and 22Rv1) had high expression levels of AR, 22Rv1 also strongly expressed the AR-V7 splice variant (lower band). However, none of these cell lines had detectable expression of ERs. In the case of human AR-negative PCa cell lines (DU145 and PC3), they both showed no expression of AR and ERβ. However, longer exposure revealed weak but detectable expression of ERα in PC3 cells.

**Figure 2:**
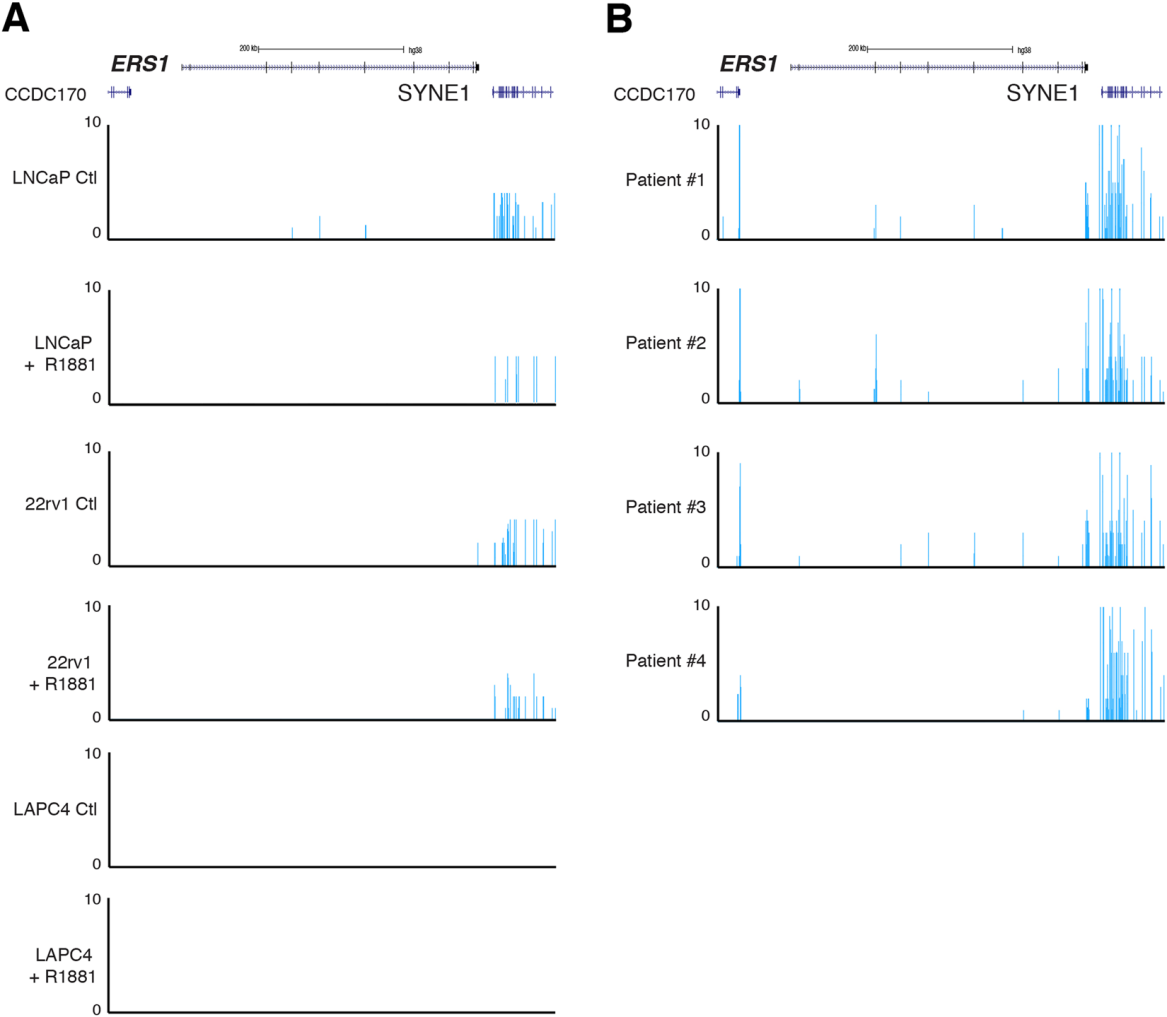
Comparison of mRNA expression of estrogen receptors between patient biopsies and PCa cell lines. (**A**) UCSC genome browser view of RNA-seq data at the *ESR1* locus in LNCaP, LAPC4, and 22Rv1 cells, with and without 24 h treatment with the synthetic androgen R1881. (**B**) UCSC genome browser view of RNA-seq data at the *ESR1* locus in 4 patients with PCa.

We also used RNA-seq data from 3 of these cell lines and from patient biopsies to analyze expression of the *ESR1* and *ESR2* genes, encoding respectively ERα and ERβ. As seen in Fig. 2A, there was no detectable expression of full length *ESR1* mRNA in LNCaP, LAPC4, and 22Rv1 cell lines, either with or without R1881 treatment. Note that the only signal observed in these cells is at *SYNE1*, a gene located close to *ESR1* (Fig. 2A). In comparison, *ESR1* mRNA was detectable at low levels in three out of four patient samples tested in RNA-seq (Fig. 2B). *ESR2* mRNA was undetectable in all our cancer cell lines and patient samples tested (data not shown).

Given the lack of detectable expression of ERα and ERβ in most PCa *in vitro* models, it is hard to reconcile with previously published results that showed decreased proliferation and survival of these cell lines following treatments with various SERMs. These studies, as reviewed in the introduction, mostly used high concentrations of SERMs, often 100 to a 1000 times over their selectivity for ERs, suggesting that the observed effects were due to off-target effects. In that context, we wanted to specifically delineate the impact of estrogens and SERMs treatment on PCa cells by using specific ERα and ERβ ligands and SERMs at concentrations specific to ERs to mimize off-target effects.

### Optimization of hormonal and SERM treatments using MCF7 cells

We first used MCF7 cells as controls to optimize treatments, as they are well-established in the breast cancer field to harbour high expression levels of ERα and no detectable expression of ERβ, and to be highly sensitive to estrogen stimulation^48^. These cells have been widely used to study the estrogen-dependent growth of breast cancer as well as to test anti-estrogen treatments. To validate that the different ERα ligand concentrations were biologically relevant, we used this model to optimize our proliferation assays using estradiol (E_2_), the ERα-specific ligand PPT, the ERβ-specific ligand DPN, five distinct SERMs that show anti-estrogen activity in breast cancer cells, and fulvestrant, a pure anti-estrogen that show antagonist functions of both receptors in all tissues tested.

**Figure 3:**
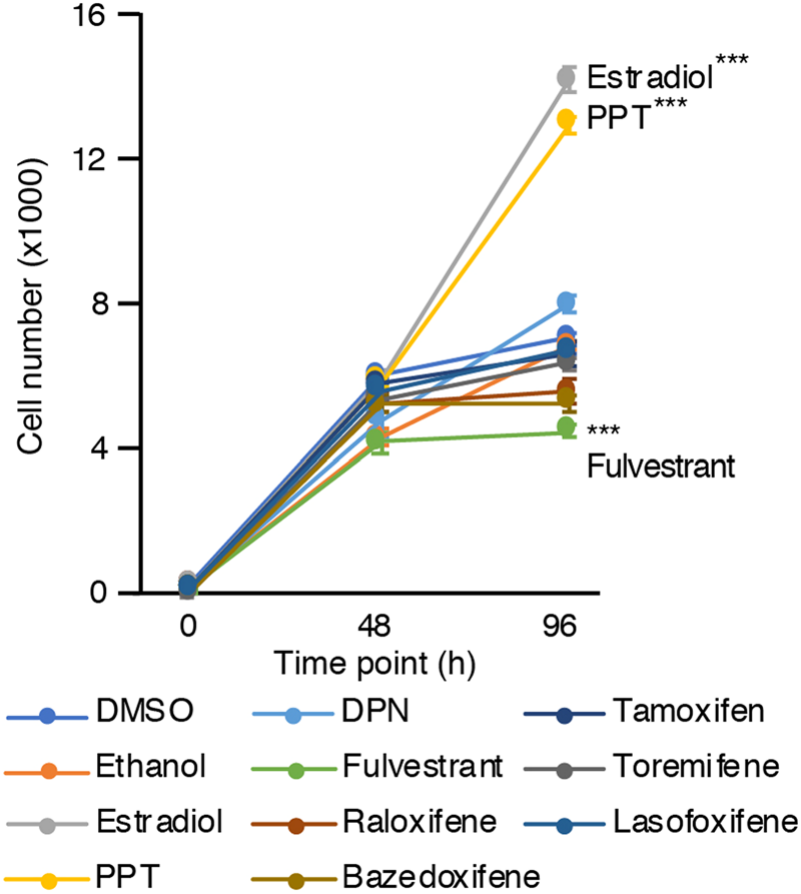
Proliferation protocol optimization using MCF7. Cell number of MCF7 after treatment with estrogenic and anti-estrogenic compounds at time point 0 h, 48 h and 96 h. Values were determined with crystal violet staining and are presented as the average ± standard error of the mean (n = 8 samples/treatment). Data from one representative experiment out of four independent experiments is shown. Asterisks (***p < 0.001) indicate that modulation of proliferation was significant in at least three out of four experiments.

In our settings, and as expected, treatment with E_2_ significantly increased MCF7 cell proliferation (Fig. 3). PPT was used at a lower concentration to avoid ERβ activation. Similar to estradiol, PPT significantly induced MCF7 proliferation rates (Fig. 3). Treatment with DPN was made at a concentration that could specifically activate the ERβ without activating ERα. As such, no significant changes in cell proliferation was noticeable for this compound.

We also treated MCF7 cells with fulvestrant. At a concentration where both receptors are blocked, 19 nM, treatment with this anti-estrogen significantly decreased cell proliferation compared to controls, probably reflecting blockade of low levels of residual estrogens in our experiment setting. In addition, five different SERMs were used, namely tamoxifen, bazedoxifene, raloxifene, toremifene, and lasofoxifene. As expected, in absence of estrogens, none of them had a significant impact on MCF7 cell proliferation.

### Treatments of AR-negative PCa cell lines

**Figure 4:**
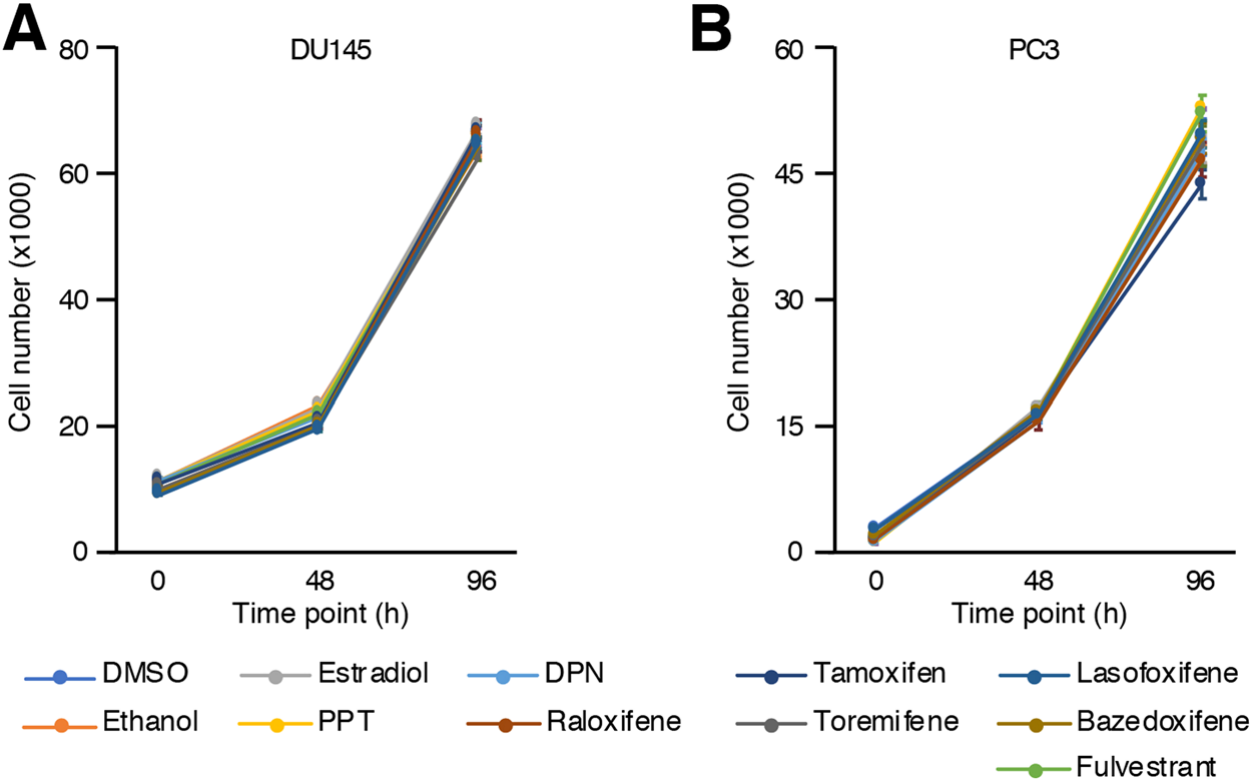
No significant modulation of proliferation upon hormonal treatment in AR-negative PCa cell lines (PC3 and DU145). Cell number of DU145 (**A**) and PC3 (**B**) cells after treatment with estrogenic and anti-estrogenic compounds detailed in the legend at time point 0 h, 48 h and 96 h. Values were determined with crystal violet staining and are presented as the average ± standard error of the mean (n = 8 samples/treatment). Data from one representative experiment out of three independent experiments is shown. There was no reproducible significant modulation of proliferation.

We then studied two AR-negative PCa cell lines, namely PC3 and DU145 cells. Contrary to MCF7 cells, E_2_ or PPT had no significant impact on DU145 cells (Fig. 4A), in line with undetectable levels of ERα in these cells. Consequently, none of the SERMs nor fulvestrant had any significant impact on cell proliferation neither. In the case of PC3 (Fig. 4B), we observed the same pattern as for DU145. None of the estrogenic compounds activating ERα (PPT), ERβ (DPN) or both (E_2_) nor the anti-estrogenic compounds had a significant impact of PC3 cell proliferation and survival after 4 days of treatments when compared to vehicles. Our results indicate that even if ERα is expressed at low levels in PC3 cells, it does not contribute to cell proliferation in standard *in vitro* culture conditions.

### Treatments of AR-positive PCa cells

Then, we studied three AR-positive PCa cell lines, namely LNCaP, LAPC4, and 22Rv1 PCa cells. They were all isolated from patients with castration-resistant PCa^63–65^. LNCaP cells have high AR expression and depend on its activity for proliferation, while LAPC4 and 22Rv1 are considered as androgen-sensitive cell lines, *i.e*. that do not depend on AR for proliferation but that are positively stimulated by androgens.

**Figure 5:**
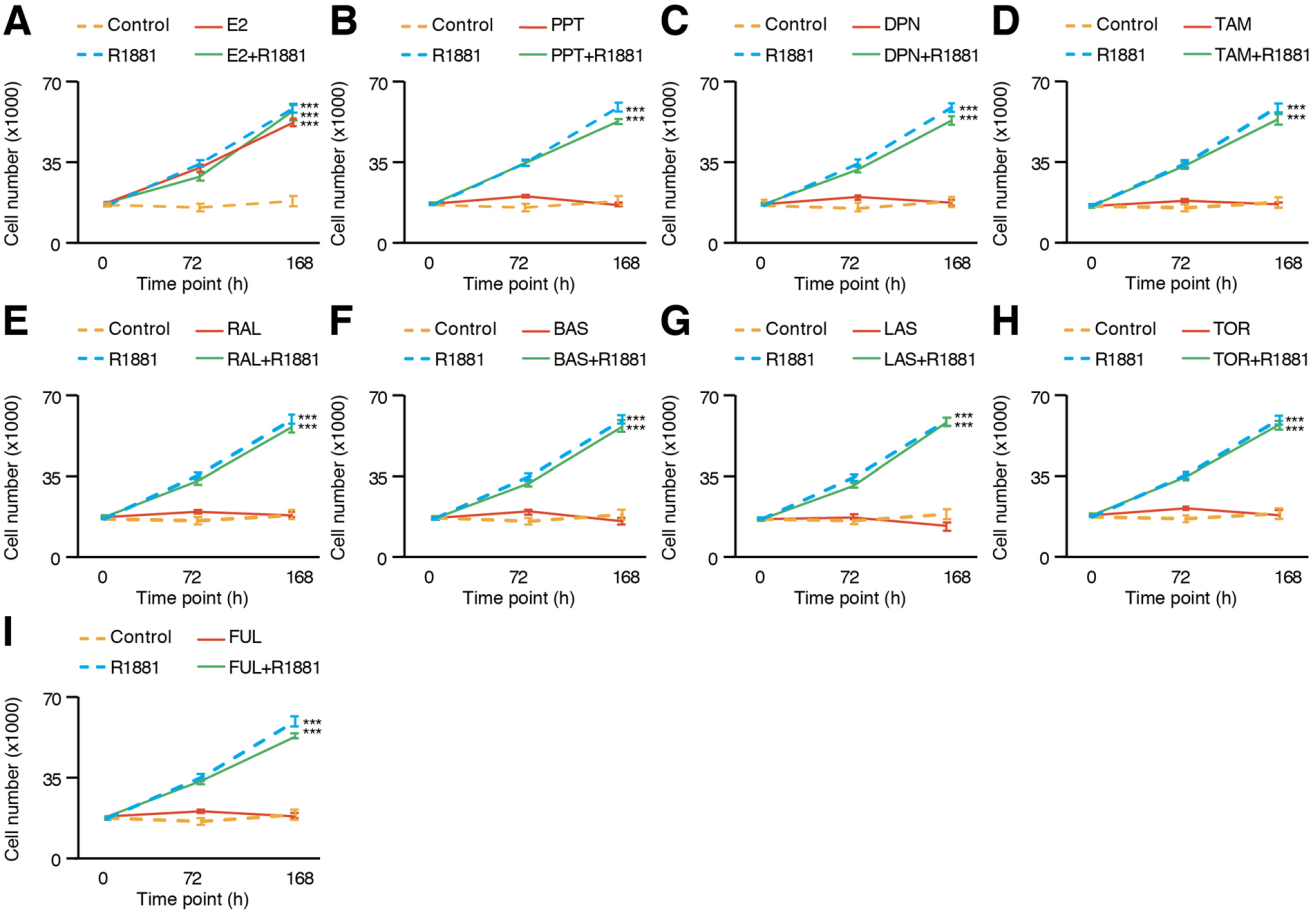
Proliferation of LNCaP cells is increased by E_2_ only in an AR-dependent manner. LNCaP cell number after treatment, with and without R1881, of either E_2_ (**A**), PPT (**B**), DPN (**C**), tamoxifen (**D**), raloxifene (**E**), bazedoxifene (**F**), lasofoxifene (**G**), toremifene (**H**), and fulvestrant (**I**). Values were determined with crystal violet staining and are presented as the average ± standard error of the mean (n = 8 samples/treatment). Data from one representative experiment out of six independent experiments is shown. Asterisks (***p < 0.001) indicate that modulation of proliferation is significant in at least four out of six independent experiments.

In absence of androgens, we observed no changes in LNCaP cell numbers, but a significant increase in proliferation after treatment with R1881 (Fig. 5A). LNCaP cells have been used in several previous studies on the effect of estrogens in PCa, even though their AR is mutated and can be activated by E_2_ besides androgens^46,47^. Thus, as expected, E_2_ significantly increased LNCaP cell proliferation compared to controls (Fig. 5A). However, no significant difference was observed when the cells were co-treated with R1881, suggesting that this response is purely AR-dependent. Consistent with this idea, treatment with specific ER agonists PPT or DPN had no significant impact on PCa cell proliferation (Fig. 5B,C). In the case of the five SERMs tested, such as tamoxifen (Fig. 5D), raloxifene (Fig. 5E), bazedoxifene (Fig. 5F), lasofoxifene (Fig. 5G), and toremifene (Fig. 5H), no significant increase or decrease of proliferation was noted after treatment, with or without the presence of androgens. After being treated with a pure antagonist of both estrogen receptors, fulvestrant, cell proliferation had once again no significant modulation (Fig. 5I) regardless of co-treatment with androgens. These results indicate that in LNCaP cells, E_2_ is solely acting through AR activation and not through modulation of ERs activity. It also suggests that these SERMs cannot modulate mutated AR activity.

**Figure 6:**
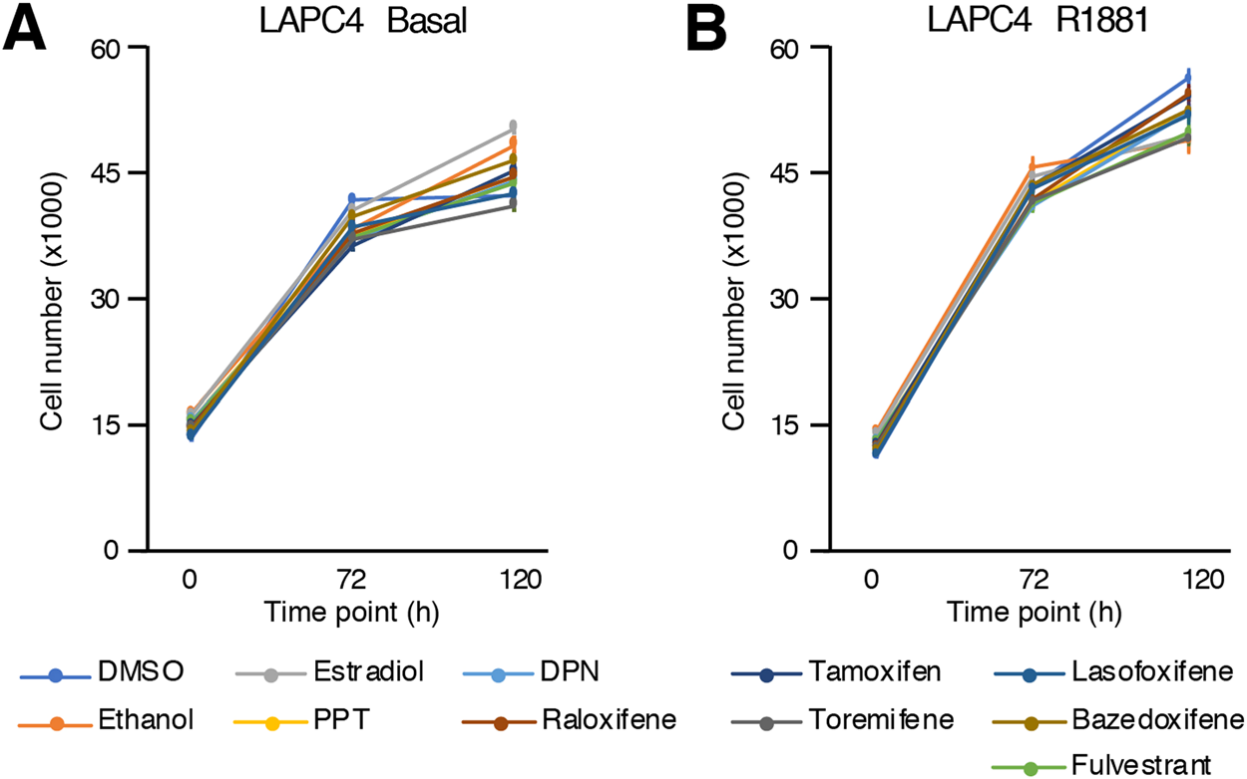
No significant modulation of proliferation upon estrogenic/anti-estrogenic treatment in LAPC4 cell number after treatment with estrogenic and anti-estrogenic compounds, without (**A**) and with co-treatment with R1881 (**B**). Values were determined with crystal violet staining and are presented as the average ± standard error of the mean (n = 8 samples/treatment). Data from one representative experiment out of six independent experiments is shown. There was no reproducible significant modulation of proliferation.

The second AR-positive cell line tested was LAPC4, a cell line that does not depend on AR for proliferation but that is still sensitive to androgens. Similar to AR-negative PCa cell lines (Fig. 4), E_2_ did not modulate significantly LAPC4 cell proliferation, and neither did PPT or DPN (Fig. 6A,B) regardless of the presence of androgens in the culture media. Consequently, treatment with the anti-estrogen fulvestrant or any SERMs did not reveal any significant impact on LAPC4 cell proliferation (Fig. 6A,B). This is consistent with lack of detectable expression of both ERs in these cells (Figs. 1 and 2A).

**Figure 7:**
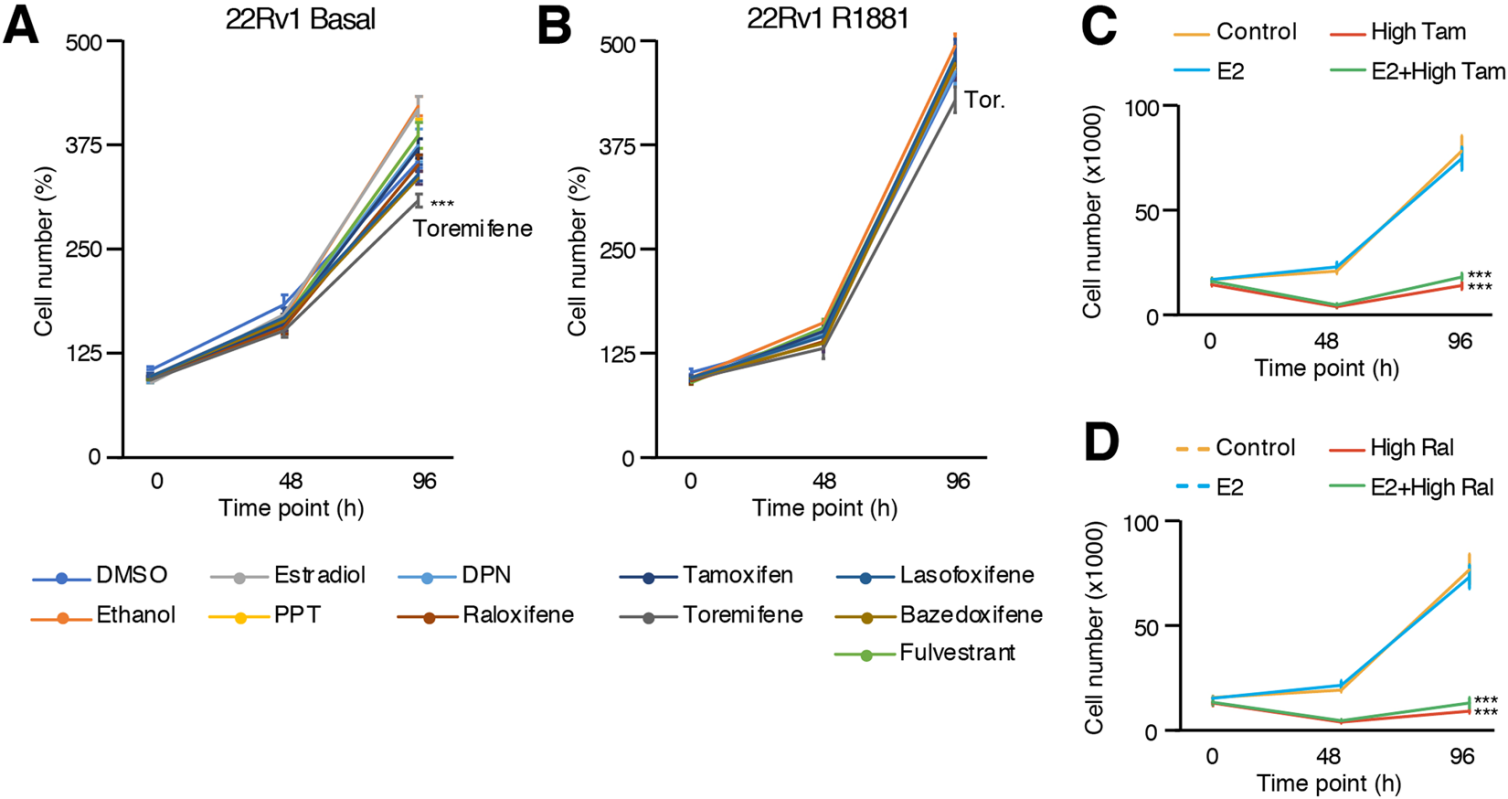
Proliferation of 22Rv1 cells is decreased with toremifene (SERM) only when androgens are absent. 22Rv1 cell number after treatment with estrogenic and anti-estrogenic compounds, without (**A**) and with co-treatment with R1881 (**B**). Values were determined with crystal violet staining and are presented as the average ± standard error of the mean (n = 8 samples/treatment). The average of two experiments out of five independent experiments is shown. 22Rv1 cell number after treatment with high concentrations of tamoxifen (**C**) and raloxifene (**D**) at 10 µM and 28.5 µM, respectively. Data from one representative experiment out of three independent experiments is shown. Asterisks (*p < 0.05, **p < 0.01, ***p < 0.001) indicate that modulation of proliferation is significant compared to controls.

We also studied the AR-positive PCa cell line 22Rv1, which is characterized by high expression of AR-V7, a splice variant of AR that lacks the ligand binding domain^66^. As for LAPC4 cells, 22Rv1 cells are not dependent on androgens for proliferation but are nevertheless sensitive to it. When treated with agonist ligands of ERs, with and without co-treatment with androgens, 22Rv1 did not have their proliferation significantly modulated (Fig. 7A,B). As for other cell lines, the anti-estrogen fulvestrant and most SERMs did not have any significant impact on 22Rv1 cell proliferation, regardless of co-treatment with R1881 (Fig. 7A,B). Interestingly, treatment with the SERM toremifene induced a small but significant decrease of 22Rv1 cell proliferation in absence of androgens (Fig. 7A), which was observed in all our independent experiments.

Finally, to study if the results obtained in previous studies were ERs-independent off-target effects due to treatments at high concentrations, we also tested high concentrations of the two most studied SERMs in *in vitro* PCa models, namely tamoxifen and raloxifene. In 22Rv1 cells, even though E_2_, PPT, and DPN had no significant impact on proliferation at physiological levels (Fig. 7A,B), treatments with tamoxifen, and raloxifene at 10 uM and 28.5 uM, respectively, significantly impaired cancer cell proliferation (Fig. 7C,D). These results demonstrate that SERMs, at high concentrations, can block PCa cell proliferation in an ERs-independent manner.

## Discussion

The goal of the current study was to perform a systematic investigation of estrogens and anti-estrogens impact on PCa cell proliferation with appropriate controls, dosages, and in multiple well-studied *in vitro* PCa models. Globally, we observed that estrogens and anti-estrogens do not have a significant impact on proliferation at dosages where these molecules bind specifically to ERα, ERβ, or both. Consistent with an absence of effect of these compounds on PCa cell proliferation, mRNA and protein expression data indicate that for the majority of these models, ERs’ expression levels are undetectable. Therefore, our study highlights that these models are inappropriate to study ERs functions in PCa cells, and further suggests the usage of alternative PCa models to properly assess the roles of the estrogen signaling pathway in PCa. Importantly, our study also indicates that most previous studies most probably reported off-target effects of SERMs in context of PCa.

Certainly the most widely used *in vitro* model of PCa, LNCaP cells have been isolated from a patient with castration-resistant PCa^63^. One of the mechanisms that explain this resistance is an AR mutation in its ligand binding domain, which allows AR to bind and be activated by other steroids, including E_2_^46,47^. Thus, studying ERs functions by using only E_2_ will inevitably lead to misinterpretations and biasing the real contribution of the estrogen signaling pathway in PCa cell proliferation^50–52^. Our results demonstrate that the significant increase of proliferation induced by E_2_ is mediated through mutated-AR activation and not through ERs activation. Indeed, the specific agonists of each estrogen receptor, PPT and DPN, do not modulate proliferation compared to E_2_, suggesting low, if any, contribution of ERs on LNCaP cell proliferation. Furthermore, it is rather clear now using validated ERα and ERβ antibodies that these cells do not express these receptors^48,53,54^. It is thus hard to reconciliate the inhibitory effect of tamoxifen and raloxifene on LNCaP cell proliferation in an ERα-dependent manner^67^, and the high dosages used, sometimes up to 10^−4^M, probably explain this inhibitory off-target effect. Results obtained with 22Rv1 cells, showing that estrogens and SERMs have no impact on proliferation at ERs-specific concentrations but decreased proliferation at high concentrations (Fig. 7), support this conclusion.

The high concentrations previously employed have most probably also led to positive effects on proliferation in other cell lines independently of ERs expression levels and activity. Indeed, LNCaP, PC3, and DU145 cells have been studied in several previous publications, using notably tamoxifen and raloxifene at high concentrations and without co-testing estrogenic positive controls such as E_2_^67–69^ to ensure proper ERs activation or inhibition through SERMs. Our results showed undetectable protein expression levels of both ERs in DU145 and LNCaP cells and weak, but detectable, levels of ERα in PC3 cells. Yet, activation of this receptor with E_2_ or PPT had no significant modulation of proliferation in the latter cell line, and neither did the anti-estrogens. In that context, we believe that inhibitory effects of tamoxifen and other SERMS reported in these models at high concentrations are mostly due to off-target effects, and not through modulation of ERs activity. In line, using high dosages of SERMs in 22Rv1 does recapitulate previous reports on decreased PCa cell proliferation, supporting off-targets effects in that context. Importantly, it was recently reported that several SERMs, such as tamoxifen, at high concentrations in the µM range, are microtubule modulators that can decrease cancer cell proliferation^70^. Such ERs-independent effects most probably explain the previously observed results. In addition, we cannot exclude that, in PC3 cells, ERα contributes to the cancer phenotype, such as in regulating invasion or migration. Overall, our study highlights the importance of using appropriate agonist ligands along with specific dosages and anti-estrogenic compounds to study more precisely the ERs activity in PCa models.

An important point raised in the last 2 years, as exemplified in the current study, is either or not ERs are expressed at all in some of the most commonly used PCa models. Indeed, there was no consensus as to whether LNCaP, DU145, and PC3 express ERβ^69,71,72^ or not^56,69,73^. Most of these discrepencies came from usage of unspecific antibodies. To counter this issue, several groups published systematic validation of ERβ antibodies^48,53,54^. Importantly, it was confirmed that LNCaP cells do not express ERβ. Yet, despite state-of-the-art validation using mass spectrometry, it is still debated if ERβ is expressed or not in the human prostate and PCa tissues^48,53,54^. Furthermore, results from genetically engineered mouse model disrupting *Esr2* are also conflictual^74,75^. A similar problem is observed for ERα expression in some PCa cell line models, notably in LNCaP cells^25,50,71,73^, although to a lesser extend due to the important work done on this receptor in the breast cancer field. The general consensus is that ERα is expressed in prostate stromal cells, as well as in human PCa and castration-resistant PCa tissues^24,34,35,76,77^. This is supported by our reanalysis of RNA-seq from PCa tumours in which *ESR1* was expressed in most samples (Fig. 2B).

The G protein-coupled estrogen receptor 1 (GPER), located at the plasma membrane and not part of the nuclear receptor family like the ERs, was not investigated in this study. Although it can also be activated by E_2_, it is probably not involved in proliferation of the PCa cell lines tested herein, at least in standard cell culture conditions. Indeed, if GPER was involved in regulating PCa cell proliferation, a significant modulation of proliferation would have been observed upon treatment with E_2_.

One interesting result we observed is the significant decrease of proliferation in 22Rv1 cells by toremifene, a SERM known to be an agonist of ERs in bone. While PCa patients follow an anti-androgenic therapy, one of the major side effects is bone loss that can lead to osteoporosis^78–80^. A few clinical trials have tested toremifene in that context, which revealed well-kept bone mineralization in patients taking this SERM combined with anti-androgen therapy^81–84^. Interestingly, the significant decrease of proliferation induced by this SERM only occurred in 22Rv1 cells, which express high levels of AR-V7. Considering that E_2_ had no significant effect on proliferation (Fig. 7) and Western blots results indicating a lack of expression of both ERs (Fig. 1), one possibility is that toremifene effect on proliferation is mediated by AR-V7. Yet, additional mechanistic studies are required to test this hypothesis.

Overall, our results demonstrate that most *in vitro* PCa models actually lack detectable expression of both ERs in standard culture conditions. Furthermore, they suggest that previous studies using these models most probably reported ERs-independent effects of SERMs due to the high concentrations used. We cannot exclude that lack of ERs expression *in vitro* is an artifact of these model systems, as ERs, and particularly ERα, are most probably expressed *in vivo* and relevant to PCa biology. In conclusion, our results indicate that classic PCa models are not appropriate to study the estrogen-signaling pathway and highlight the necessity to find appropriate PCa models that express biologically relevant levels of ERα and ERβ.

## Materials and Methods

### Cell culture

Six cell lines were used for proliferation assays: one breast cancer cell line serving as an ERα positive control (MCF7 [RRID: CVCL_0031]), two AR-negative PCa cell lines (PC3 [RRID: CVCL_0035] and DU145 [RRID: CVCL_0105]) and three AR-positive PCa cell lines (LNCaP [RRID: CVCL_1379], 22Rv1 [RRID: CVCL_1045], LAPC4 [RRID: CVCL_4744]). All of them were initially obtained from ATCC, were kept in culture for no more than 3 months after resuscitation and were tested every 4 months for mycoplasma presence. They were grown in phenol red RPMI media supplemented with 10% fetal bovine serum (FBS), penicillin, streptomycin, and sodium pyruvate, and kept in incubators at 37 °C and 5% CO_2_. The media was changed every two days and confluence in plates was kept below 75%.

### Western blots

To evaluate the protein expression of the receptors, cell lysates of all cell lines were harvested in Buffer K supplemented with protease and phosphatase inhibitors as previously described^5^ before analysis by Western Blots. The primary antibodies used were to detect α-tubulin (11H10 [RRID: AB_10695471], Cell Signaling Technology, dilution 1:1,000), AR (441 [RRID: AB_626671], Santa Cruz Biotechnology, dilution 1:1,000), ERα (F-10 [RRID: AB_627558], Santa Cruz Biotechnology, dilution 1:1,000) and ERβ (CWK-F12 [RRID: AB_2722105], DSHB, dilution 1:1,000). Complete Western Blot figures are shown in Supplemental Fig. S1.

### RNA-seq analysis

RNA-seq data we previously generated were used to study mRNA expression of ERα, encoded by the *ERS1* gene in LNCaP and LAPC4 cells^85^. Briefly, cells were seeded for 48 h in RPMI 1640 media without phenol red and with 5% CSS for steroid deprivation. They were then treated for 24 h with 10 nM R1881 or vehicle (96% EtOH) before being harvested for RNA purification and sequencing. We used the same protocol for 22Rv1 cells and after sequencing they were processed as described previously to obtain transcripts per million (TPM) values. In short, FastQC was used for raw sequencing data quality control^86^ and Trimmomatic was used to trim adaptor and over-represented sequences^87^. Pseudoalignement to the transcriptome (hg38) was then performed using Kallisto^88^ and samples were visualized using the UCSC genome browser. Results were then converted to bedgraphs before being loaded in the UCSC genome browser for visualization.

### Hormonal treatments

Cells were firstly seeded in 96-well plates with phenol red-free RMPI 1640 supplemented with 5% charcoal-stripped serum (CSS) and incubated for 48 h to allow steroid deprivation. Media was then renewed (100 μL per well) and treatment was added. The chosen treatment concentration for estrogenic/anti-estrogenic compounds and SERMs correspond to 5-fold the EC_50_/IC_50_ specific to each compound, except for tamoxifen and toremifene (see Table 1 for more details). The compounds used for treatments were: DMSO (vehicle; Sigma), ethanol 96% (vehicle; Greenfield Global), 17β-estradiol (10 nM; Sigma), PPT (1,3,5-tris(4-hydroxyphenyl)-4-propyl-1H-pyrazole; Santa Cruz Biotechnology), DPN (2,3-bis(4-hydroxyphenyl) propionitrile; Santa Cruz Biotechnology), fulvestrant (ICI 182,780; Santa Cruz Biotechnology), 4-OH-tamoxifen (Santa Cruz Biotechnology), raloxifene hydrochloride (Tocris), toremifene citrate (Santa Cruz Biotechnology), bazedoxifene (Tocris) and lasofoxifene (Santa Cruz Biotechnology). AR-positive PCa cell lines were also co-treated or not with the synthetic androgen R1881 (10 nM; Steraloids). The media and treatments were renewed every 48 h until the end of the proliferation assays (either after 96 h or 168 h in total, depending on the cell lines).

### Quantification of cellular proliferation with crystal violet

The crystal violet assay developed by Feoktistova *et al*.^89^ was used to stain the cells in order to determine cell number after treatment. Media was firstly removed, then 50 μL of a 0.5% crystal violet solution was added in each well. The plates were left on a rocking bench for 30 mins at room temperature, rinsed thoroughly 4 times with water, and left to dry at least 48 h (protected from light). The stained cells were then lysed by adding 200 μL of SDS 2% in each well, and the plates were left on a rocking bench for 4 h at room temperature. The plates had their optical density (OD) measured at 570 nm using a spectrophotometer. OD values were converted into cell numbers by using standard curves specific to each cell line.

### Quantification of cellular proliferation with CyQUANT

Given weaker adherence of LAPC4 and LNCaP cells, the CyQUANT Cell Proliferation Assay Kit (Catalog #: C7026, Invitrogen) was also used to confirm proliferation assays. After treatment, media was removed from wells, then the plates were frozen at −20 °C at least 24 hours before analysis using the manufacturer’s instructions. The plates were then thawed, and a solution of CyQUANT was added in each well containing cells. The plates were incubated in the dark for 5 minutes, and the fluorescence was measured at 480 nm of excitation and 520 nm of emission using a spectrophotometer. Fluorescence values were converted into cell numbers by using standard curves specific for each cell line.

### Statistical analysis for proliferation assays

Proliferation assays were done at least 3 times independently for each cell line, with 4–8 biological samples per group per experiment. To test for statistical significance of estrogenic, anti-estrogenic, and androgenic treatments, one-way ANOVA with Tukey HSD and Dunnett’s *post-hoc* tests were performed using XL STATS.

## Supplementary information

Supplementary information.

## Data Availability

RNA-seq data generated during this study is available from the Gene Expression Omnibus (GSE128749 for LNCaP and LAPC4 cells; GSE128201 for 22 Rv1) and are described elsewhere^85^. Data from biopsies come from SRA access number SRR1164789, SRR1164790, SRR1164791 and SRR1164792^90^.
