# Supplementary figures and images for: A Systematic Study of the Impact of Estrogens and Selective Estrogen Receptor Modulators on Prostate Cancer Cell Proliferation
Source: Sci Rep. 2020 Mar 4;10:4024. doi: 10.1038/s41598-020-60844-3 (PMC7055213; doi:10.1038/s41598-020-60844-3)

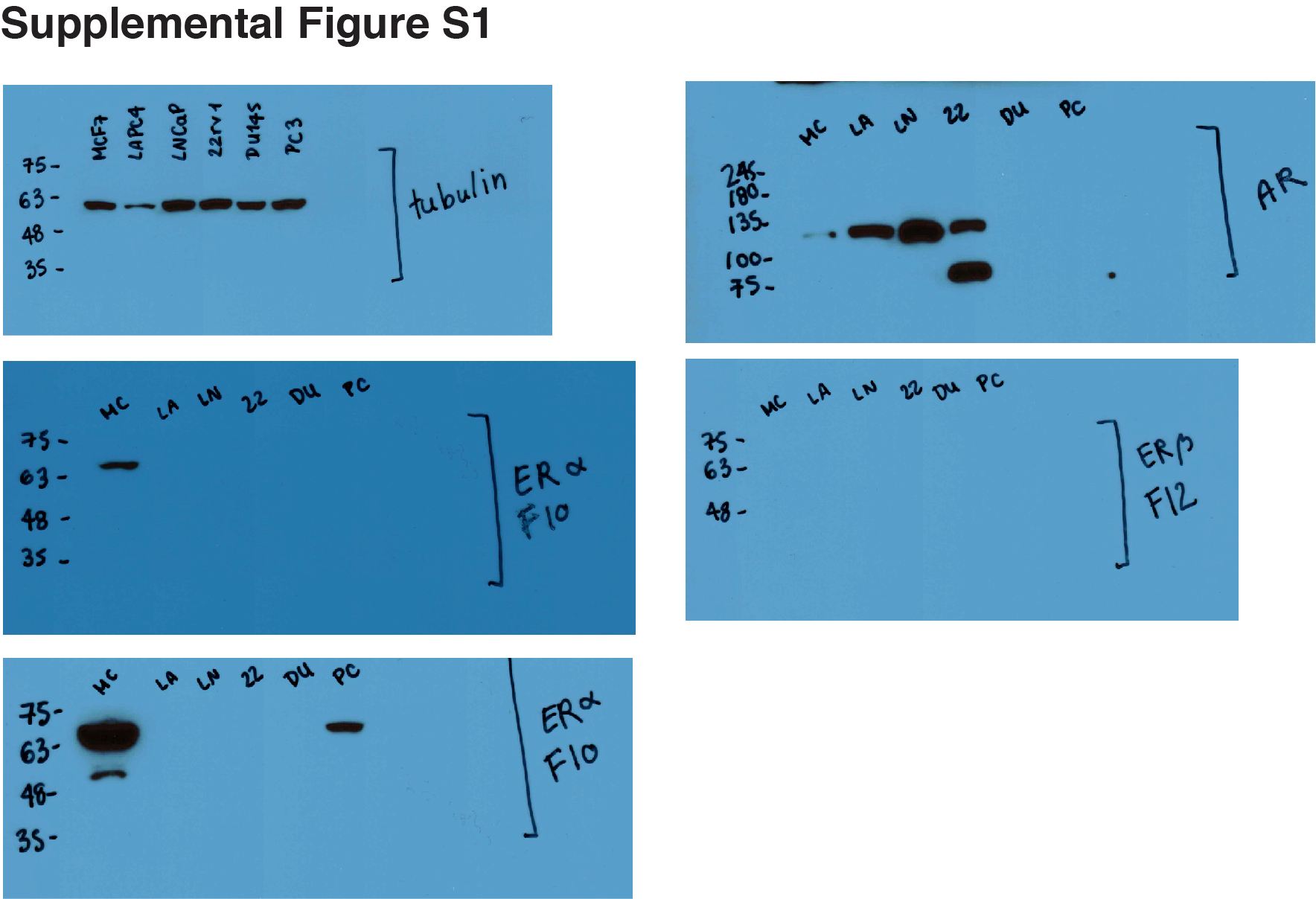

Supplement: Supplementary file 1 — Supplementary information. [file 41598_2020_60844_MOESM1_ESM.jpg]
